# Supplementary material for: Alleviating Heavy Metal Toxicity in Milk and Water through a Synergistic Approach of Absorption Technique and High Voltage Atmospheric Cold Plasma and Probable Rheological Changes
Source: Biomolecules. 2022 Jun 29;12(7):913. doi: 10.3390/biom12070913 (PMC9312926; doi:10.3390/biom12070913)
Supplement: Supplementary file 1 [file biomolecules-12-00913-s001.zip › biomolecules-1778533-supplementary.pdf]

Supplemental material

The LVR determination provides a dynamic mechanical report of the change in the microstructure under a critical strain.

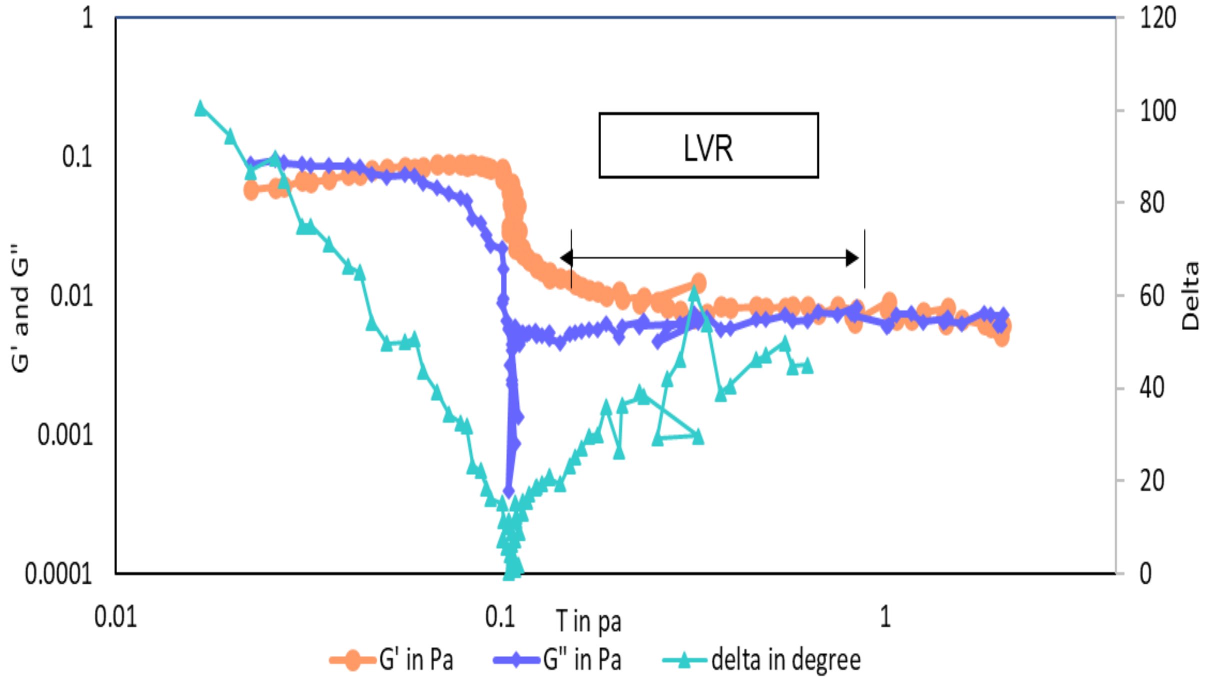

Figure S1. Linear Viscoelastic Region of the milk samples.

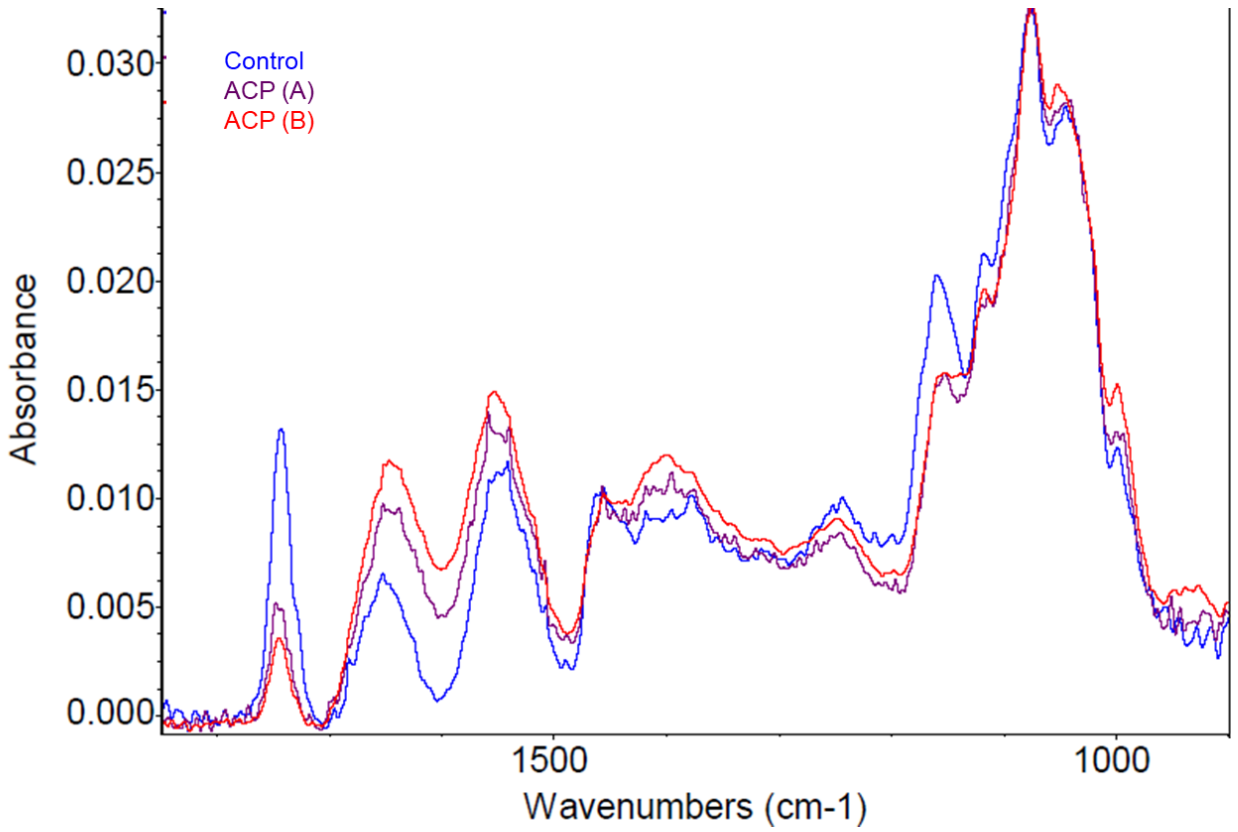

Figure S2: Expanded fingerprint region from FTIR spectrum of the milk samples. ACP (A) and ACP (B) denotes the sample treated with 50 kV for 2 minutes with 0- and 24-hours storage time respectively

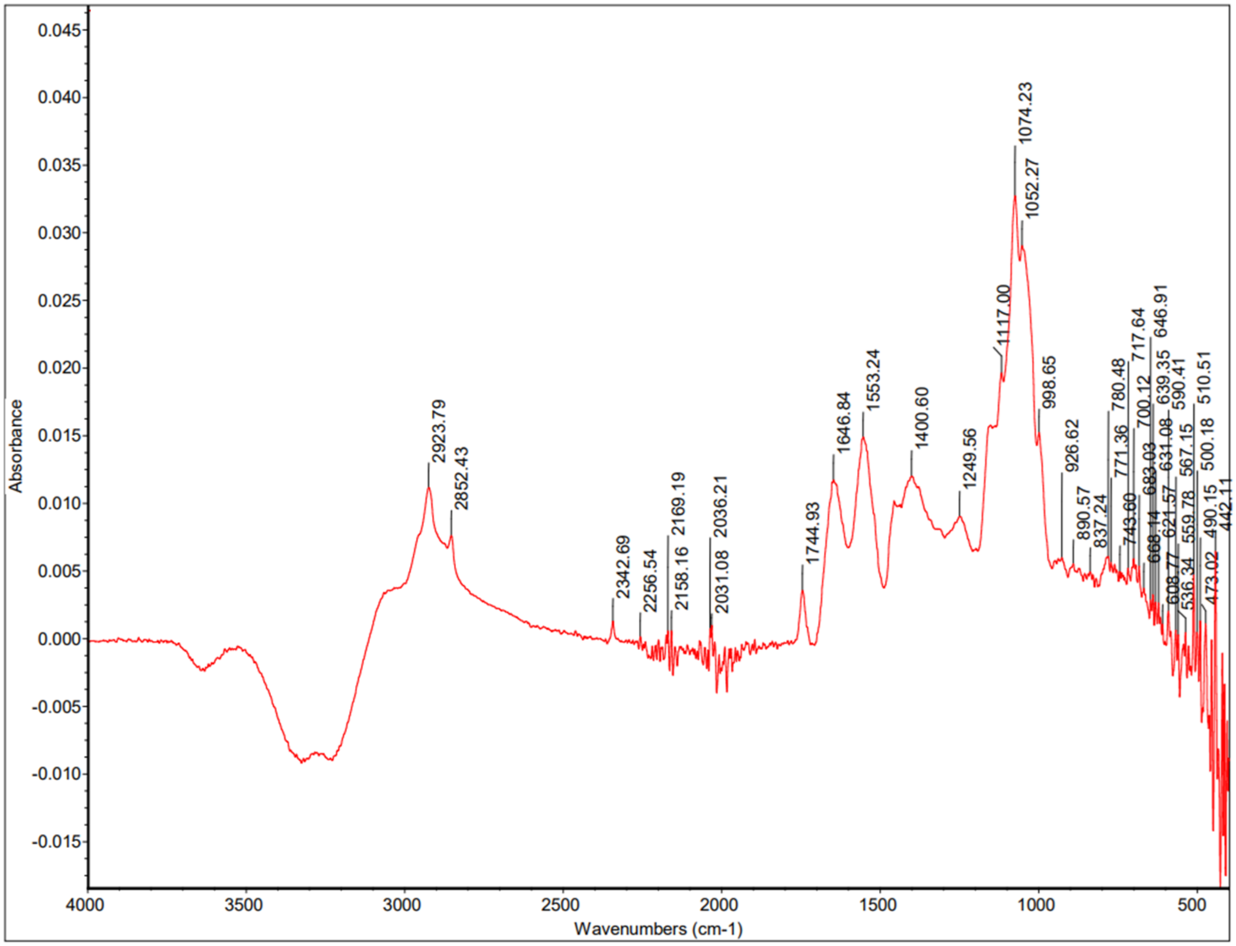

Figure S3: Peaks from the FTIR spectrum of the milk samples when the sample was plasma treated with 50 kV for 2 minutes with 24-hours storage time.
